# Neuropsychologische Profile und Vulnerabilität für psychische Störungen bei ausgewählten genetischen Syndromen mit Störungen der Intelligenzentwicklung

**DOI:** 10.1007/s00115-026-01962-7

**Published:** 2026-04-14

**Authors:** Klaus Sarimski

**Affiliations:** 1 Pädagogische Hochschule Heidelberg, Heidelberg, Deutschland; 2 Löfftzstr. 5, 80637 München, Deutschland

**Keywords:** Kognition, Exekutive Funktion, Down-Syndrom, Fragiles-X-Syndrom, Prader-Willi-Syndrom, Cognition, Executive function, Down syndrome, Fragile X syndrome, Prader-Willi syndrome

## Abstract

Die Analyse von individuellen kognitiven Profilen und die Beurteilung der Teilkomponenten von exekutiven Funktionen trägt zu einem besseren Verständnis der Probleme bei, die Kinder, Jugendliche und Erwachsene mit intellektueller Beeinträchtigung bei der Bewältigung von sozialen Anforderungen haben. Charakteristische Profile und Auffälligkeiten in den exekutiven Funktionen beim Down‑, Fragilen-X- und Prader-Willi-Syndrom werden geschildert. An diesen Beispielen lassen sich Zusammenhänge zu psychischen Auffälligkeiten, auch als Verhaltensphänotypen bezeichnet, bei Kindern, Jugendlichen und Erwachsenen mit diesen genetischen Syndromen illustrieren.

## Abstract (en)

The analysis of individual cognitive profiles and the assessment of executive function components contributes to a better understanding of the problems that children, adolescents, and adults with intellectual disabilities experience in coping with social demands. Characteristic profiles and abnormalities in executive functions in Down syndrome, fragile X syndrome, and Prader–Willi syndrome are described. These examples illustrate associations with psychological problems—so-called behavioral phenotypes—in children, adolescents, and adults with these genetic syndromes.

Wie schon die diagnostische Kategorie *Intellektuelle Beeinträchtigungen* der 5. Auflage des Diagnostic and Statistical Manual of Mental Disorders (DSM‑5; [1]) wird die diagnostische Kategorie *Störungen der Intelligenzentwicklung* der 11. Auflage der International Classification of Diseases (ICD-11; [15]) nicht allein durch das Defizit des allgemeinen Intelligenzniveaus (Intelligenzquotient, IQ) als einziges Merkmal definiert, sondern durch das zweite Merkmal, die Beeinträchtigungen des adaptiven Verhaltens (Defizite kognitiver, sozialer oder alltagspraktischer Fähigkeiten). In ihnen spiegelt sich die Beeinträchtigung einzelner neuropsychologisch definierter Fähigkeiten und exekutiver Funktionen wider, die den kognitiven Prozessen zugrunde liegen. Diese differenzierte Analyse von individuellen kognitiven Profilen und die Beurteilung der Teilkomponenten von exekutiven Funktionen, die für die Bewältigung von sozialen Anforderungen relevant sind, wird dem Anspruch, die Fähigkeiten und Unterstützungsbedürfnisse von Kindern, Jugendlichen und Erwachsenen mit intellektueller Beeinträchtigung zu verstehen, besser gerecht als ein eindimensionales Konzept, wie es sich in einem globalen Intelligenzquotienten niederschlägt [2]. Diesbezügliche Beeinträchtigungen spielen im Bedingungsgefüge von Verhaltensauffälligkeiten eine maßgebliche Rolle.

## Differenzierung von kognitiven Profilen und exekutiven Funktionen

Die Differenzierung von kognitiven Profilen und die Beschreibung von exekutiven Funktionen erfordert differenzierte Untersuchungsverfahren. Die Auswahl von Testverfahren zur Beschreibung der Struktur der intellektuellen Fähigkeiten kann sich dabei an der Cattell-Horn-Carroll-Theorie (CHC-Theorie) orientieren, bei der 8 breite, empirisch definierte Fähigkeitsbereiche unterschieden werden, u. a. das Kurzzeitgedächtnis, Langzeitgedächtnis, die visuell-räumliche Verarbeitung, auditive Verarbeitung und die Geschwindigkeit der Informationsverarbeitung. Ein Testverfahren, das diesem Anspruch bei Kindern und Jugendlichen gerecht wird, ist z. B. die Kaufman-Assessment Battery for Children (K-ABC-II). Im neuropsychologischen Profil, das sich mit diesem Verfahren auf der Basis der Cattell-Horn-Carroll(CHC)-Theorie erstellen lässt, lassen sich dann individuelle Stärken und Schwächen von Kindern, Jugendlichen und Erwachsenen erkennen.

Exekutive Funktionen werden als übergeordnete Fähigkeiten verstanden, auf die ein Individuum für die Auswahl und das Monitoring von einzelnen Handlungsschritten bei der Bewältigung von Anforderungen zurückgreift. Dazu gehören

- die Hemmung von impulsiven Reaktionen (Inhibition),
- die kognitive Umstellungsfähigkeit,
- die Fähigkeit zur kurzzeitigen Speicherung von Informationen im Arbeitsgedächtnis und
- die Planung von Handlungsschritten.

Schwierigkeiten in exekutiven Funktionen haben erhebliche Auswirkungen auf die Alltagsbewältigung in verschiedenen sozialen Kontexten ebenso wie auf den Erfolg schulischer Lernprozesse.

Grundsätzlich können exekutive Funktionen mit spezifischen, neuropsychologisch fundierten Testaufgaben oder durch die Befragung von Bezugspersonen zu ihren Beobachtungen des Verhaltens einer Person im Alltag beurteilt werden. Zu den spezifischen Testaufgaben gehört z. B. der Wisconsin Card Sorting Test, bei dem Karten nach bestimmten, im Laufe der Bearbeitung wechselnden Regeln sortiert werden müssen, oder der Test Tower of Hanoi, bei dem unterschiedlich große Scheiben auf 3 Stäben nach vorgegebenen Regeln arrangiert werden müssen, um eine bestimmte Anordnung zu erreichen.

Es bestehen allerdings Zweifel, ob die Ergebnisse, die mit solchen Testaufgaben unter störungsfreien Bedingungen gewonnen werden, valide sind, um Aussagen über Beeinträchtigungen der exekutiven Funktionen im Alltag zu treffen. Als Alternative bietet sich z. B. das Behavioral Rating Inventory of Executive Function (BRIEF) an. Es ist für verschiedene Altersgruppen normiert, auch in deutscher Sprache zugänglich und bei Kindern und Erwachsenen mit intellektueller Beeinträchtigung validiert.

Personen mit intellektueller Beeinträchtigung erweisen sich in Untersuchungen zur Struktur ihrer intellektuellen Fähigkeiten und der Ausbildung von exekutiven Funktionen nicht als homogene Gruppe. Vielmehr lassen sich individuell unterschiedliche Profile von kognitiven und exekutiven Funktionen beschreiben, die z. T. mit der genetischen Ursache der Entwicklungsstörung assoziiert, d. h. charakteristisch für den Verhaltensphänotyp eines spezifischen Syndroms sind [22, 23]. Dies soll in diesem Beitrag beispielhaft anhand des Down-Syndroms, Fragilen-X-Syndroms und Prader-Willi-Syndroms gezeigt werden.

## Vulnerabilität für spezifische psychische Auffälligkeiten

Das Konzept der Verhaltensphänotypen richtet sich nicht nur auf die Beschreibung syndromspezifischer Fähigkeitsprofile, sondern ebenso auf die Erfassung von Verhaltensmerkmalen und psychischen Auffälligkeiten, die bei Personen mit einem bestimmten genetischen Syndrom häufiger oder stärker ausgeprägt sind als bei intellektueller Beeinträchtigung anderer Ursache [6, 22]. In diesem Beitrag soll auch erörtert werden, inwieweit spezifische Defizite in kognitiven oder exekutiven Funktionen die Bewältigung von sozialen Anforderungen erschweren und damit als biologische Disposition zu Stress und erhöhter Vulnerabilität zur Ausbildung von psychischen Auffälligkeiten beitragen können. Entsprechende Befunde zu Zusammenhängen zwischen neurokognitiven Funktionen und psychischen Auffälligkeiten liegen z. B. für Kinder mit leichter intellektueller Beeinträchtigung (unspezifischer Ursache) vor [21].

Ein Verständnis solcher Zusammenhänge ist angesichts der hohen Prävalenz von Verhaltensauffälligkeiten und psychischen Störungen bei intellektueller Beeinträchtigung von besonderer klinischer Bedeutung. Eine neuere Metaanalyse ermittelt eine Prävalenz von 38–49 % behandlungsbedürftiger Auffälligkeiten in 19 Studien, die bei Kindern und Jugendlichen mit intellektueller Beeinträchtigung durchgeführt wurden [3]. Mazza et al. [17] berichteten in ihrer Metaanalyse eine Prävalenz von 33 % für psychische Störungen bei Adoleszenten und Erwachsenen mit intellektueller Beeinträchtigung.

## Down-Syndrom

Für das Fähigkeitsprofil von Kindern, Jugendlichen und Erwachsenen mit Down-Syndrom ist eine relative Schwäche in der Aufmerksamkeit und Speicherfähigkeit für verbale Informationen, in der Inhibition der Reaktion auf irrelevante Informationen sowie in der Fähigkeit zur kognitiven Umstellung auf wechselnde Anforderungen und der Fähigkeit zur Planung von Handlungsschritten – insbesondere wenn es sich um sprachliche Instruktionen handelt – charakteristisch [11].

In einer Metaanalyse von 26 Studien, bei denen Vergleiche mit Kindern, Jugendlichen oder Erwachsenen mit gleichem mentalem Entwicklungsalter und unbeeinträchtigter Entwicklung oder mit intellektueller Beeinträchtigung anderer Ursache (z. B. Fragiles-X-Syndrom, Williams-Beuren-Syndrom) vorgenommen wurden, ermittelten Godfrey und Lee [10] über alle Altersgruppen hinweg für die Speicherung verbaler Informationen im Arbeitsgedächtnis einen sehr großen Unterschied (Effektstärke d = −1,40). Personen mit Down-Syndrom schnitten bei solchen Aufgaben wesentlich schlechter ab. Hinsichtlich der Speicherung von visuellen Informationen erwies sich der Effekt zwar ebenfalls als signifikant, aber als wesentlich kleiner (Effektstärke d = −0,32). Sowohl die auditive als auch die visuelle Merkfähigkeit entwickeln sich beim Down-Syndrom offenbar langsamer als bei Gleichaltrigen mit anderen Formen intellektueller Beeinträchtigung. Die visuell-räumliche Speicherfähigkeit scheint jedoch nicht stärker beeinträchtigt als angesichts des mentalen Entwicklungsalters zu erwarten wäre.

Tungate und Conners [27] legten eine Metaanalyse von 57 Studien vor, die zu exekutiven Funktionen bei Kindern, Jugendlichen und Erwachsenen mit Down-Syndrom durchgeführt wurden. Bei diesen Studien wurden spezifische neuropsychologisch fundierte Testaufgaben verwendet. Die Autoren fanden signifikante Unterschiede zu Vergleichsgruppen mit gleichem mentalem Entwicklungsalter und unbeeinträchtigter Entwicklung bei allen exekutiven Funktionen, d. h. der Inhibition, der flexiblen Umstellungsfähigkeit, der Funktion des Arbeitsspeichers und der Fähigkeit zur Planung und Kontrolle von Handlungen.

Auch in Studien, die über die Befragung von Eltern oder Lehrkräften berichten, zeigten sich ausgeprägte Schwächen in der Fähigkeit zur Hemmung impulsiver Reaktionen, in der flexiblen Umstellung der Aufmerksamkeit sowie – weit über das Niveau, das aufgrund des Entwicklungsalters zu erwarten wäre, hinausgehend – vor allem im Bereich der Planungsfähigkeiten [9].

Psychische Auffälligkeiten sind bei Kindern und Jugendlichen mit Down-Syndrom insgesamt zwar seltener als bei anderen Formen intellektueller Behinderung, aber häufiger als bei Kindern, bei denen keine intellektuelle Beeinträchtigung vorliegt. Aufmerksamkeits-Hyperaktivitäts-Symptome und oppositionelle Verhaltensstörungen, ritualisierte Verhaltensweisen sowie Schwierigkeiten, sich an neue Situationen anzupassen, werden im Kindesalter häufiger berichtet als bei Gleichaltrigen [18]. Bei Erwachsenen mit Down-Syndrom sind die Prävalenzraten von Ängsten, zwanghaften Störungen und Depressionen höher als bei anderen Formen intellektueller Beeinträchtigung [20].

Oppositionelle Verhaltensweisen (z. B. in Form der Missachtung von Regeln oder des Weglaufens) lassen sich in Zusammenhang bringen mit Schwierigkeiten bei der Hemmung impulsiver Handlungen, die sich bei der Analyse der exekutiven Funktionen zeigen. Die Neigung zu ritualisierten Verhaltensweisen und eine gewisse Sturheit als Charaktermerkmal lassen sich als Ausdruck von Schwierigkeiten in der kognitiven Umstellung verstehen. Sie sind mit der Ausprägung von Ängsten assoziiert, d. h.: Es scheint sich um einen adaptiven Versuch der Kinder, Jugendlichen und Erwachsenen zu handeln, Ängste in Situationen, die sie zu überfordern drohen, durch den Rückzug auf stereotype Verhaltensweisen zu reduzieren [12, 26].

## Fragiles-X-Syndrom

Auch das Profil kognitiver Funktionen bei Jungen – und mit schwächerer Ausprägung, aber mit ähnlichem Muster auch Mädchen – mit Fragilem-X-Syndrom weist spezifische Merkmale auf. In Intelligenztests zeigen sich relative Stärken bei der simultanen Verarbeitung von Informationen auf (z. B. beim Erfassen von visuellen Analogien) und beim Abruf von Wissen aus dem Langzeitgedächtnis, während sequentielle Verarbeitungsprozesse, bei denen mehrere Schritte zur Bewältigung der Aufgaben nacheinander geplant und kontrolliert werden müssen, deutlich schwerer fallen.

Schmitt et al. [24] legten eine Übersicht über die Ergebnisse von mehr als 50 Studien zu kognitiven Prozessen bei Fragilem-X-Syndrom vor. Im Vergleich zu Kindern gleichen Lebensalters oder mentalen Entwicklungsalters, bei denen keine intellektuelle Beeinträchtigung vorliegt, haben Jungen mit Fragilem-X-Syndrom mehr Schwierigkeiten in der Hemmung impulsiver Reaktionen, in der kognitiven Umstellungsfähigkeit und flexiblen Aufmerksamkeitssteuerung sowie der Fähigkeit zur Planung von Problemlöseprozessen. Schwierigkeiten in der flexiblen Ausrichtung der Aufmerksamkeit und Defizite in der Kontrolle über impulsive Verhaltenstendenzen zeigen sich auch in Studien, bei denen Jungen mit Fragilem-X-Syndrom mit Kindern mit intellektuellen Beeinträchtigungen anderer Ursache (z. B. Down-Syndrom, Prader-Willi-Syndrom) vergleichen wurden (u. a. [19]).

Auch die Speicherung von Informationen im Arbeitsgedächtnis gelingt ihnen schlechter als Kindern gleichen mentalen Alters, bei denen keine Entwicklungsbeeinträchtigung vorliegt, und schlechter als Kindern mit intellektueller Beeinträchtigung unspezifischer Ursache. Der Unterschied fällt besonders deutlich aus, wenn zusätzliche kognitive Operationen über den reinen Abruf von Operationen hinaus erforderlich sind. Bei der Planung von Problemlöseprozessen, die z. B. mit dem Tower-of-Hanoi-Test untersucht werden, haben Jungen mit Fragilem-x-Syndrom ebenfalls mehr Schwierigkeiten als jüngere Kinder mit gleichem mentalem Entwicklungsalter [13].

Eine systematische Übersicht über mehr als 25 Studien zu psychischen Störungen, Verhaltensauffälligkeiten und zur Entwicklung von sozialen Kompetenzen bei Kindern, Jugendlichen und Erwachsenen mit Fragilem-X-Syndrom liegt von Cregenzan-Royo et al. vor [5]. Die häufigsten Verhaltensauffälligkeiten, die in diesen Studien genannt werden, sind Aufmerksamkeits- und Hyperaktivitätsprobleme sowie eine ausgeprägte soziale Ängstlichkeit verbunden mit einer ausgeprägten Überempfindlichkeit gegen sensorische Reize und einer Neigung zu Stereotypien. Die motorische Unruhe ist im jüngeren Kindesalter am stärksten ausgeprägt und nimmt danach etwas ab, während Aufmerksamkeitsprobleme unverändert bleiben.

Ausgeprägte Symptome einer Aufmerksamkeits‑/Hyperaktivitätsstörung und repetitive sowie stereotype Verhaltensweisen sind bei bis zu 85 % der Jungen (und jungen Männern) mit Fragilem-X-Syndrom zu beobachten [16]. Eine soziale Angststörung wird bei 40–60 % diagnostiziert. Diese Störungen sind signifikant häufiger als bei Kindern und Jugendlichen mit Down-Syndrom, anderen Syndromen mit genetischer Ursache oder bei Kindern mit intellektueller Beeinträchtigung unklarer Genese im gleichen Alter und mit gleichem nonverbalem Intelligenzniveau [7].

Solche Auffälligkeiten im Verhalten und Erleben der Jungen mit Fragilem-X-Syndrom lassen sich im Kontext des spezifischen Profils der exekutiven Funktionen verstehen. Probleme in der Kontrolle impulsiver Reaktionen gehören zu den Kernsymptomen einer Aufmerksamkeits‑/Hyperaktivitätsstörung. Probleme in der kognitiven Umstellungsfähigkeit bedeuten Schwierigkeiten, sich auf Abweichungen von der gewohnten Routine einzustellen und die Aufmerksamkeit von sozialen Reizen (z. B. der im Gesicht des Gegenübers erkennbaren Erwartungshaltung, die als Stress erlebt wird) abzuwenden. Für Jungen mit Fragilem-X-Syndrom ist es besonders schwer, die mit solchen sozialen Anforderungen verbundenen Emotionen zu regulieren [4]. Eine ausgeprägte Verunsicherung im Umgang mit Unsicherheit (mangelnder Vorhersagbarkeit von Abläufen) spiegelt sich dann in stereotypen Verhaltensweisen (z. B. Wedeln mit den Armen) oder leichten Selbstverletzungen (z. B. Beißen in die eigene Hand) wider, die für den Verhaltensphänotyp beim Fragilen-X-Syndrom charakteristisch sind.

## Prader-Willi-Syndrom

Das kognitive Fähigkeitsprofil von Kindern, Jugendlichen und Erwachsenen mit Prader-Willi-Syndrom ist durch relative Stärken in visuellen Verarbeitungsprozessen gekennzeichnet. Dies zeigt sich sowohl in Untersuchungen mit differenzierten Intelligenztests, bei denen ihnen die Bearbeitung von Aufgaben zum Nachbauen von Mustern oder Erfassen von Analogien relativ gut gelingt, als auch im Alltag, wenn sie mit großem Geschick Puzzleteile zusammenfügen [28].

Bei Aufgaben zur Beurteilung von exekutiven Funktionen zeigen Kinder mit Prader-Willi-Syndrom mehr Schwierigkeiten mit der flexiblen Umstellung der Aufmerksamkeit und der Kontrolle impulsiver Reaktionen als Kinder mit unbeeinträchtigter Entwicklung; diese Funktionen sind auch (noch) stärker beeinträchtigt als bei Jungen mit Fragilem-X-Syndrom [28]. Bei der Befragung von Eltern mit dem Behavior-Rating-Inventory-of-Executive-Function(BRIEF)-Fragebogen liegen die Werte für Probleme in der kognitiven Umstellungsfähigkeit und Kontrolle über Handlungsschritte in fast allen Fällen im klinisch auffälligen Bereich [14]. Bei Erwachsenen zeigen sich Defizite in der Planungsfähigkeit bei komplexen Handlungen, die weit über das Maß hinausgehen, das vom mentalen Entwicklungsalter zu erwarten wäre [8].

Auch bei Personen mit Prader-Willi-Syndrom wird eine hohe Prävalenz von Verhaltensauffälligkeiten und psychischen Störungen berichtet [25]. Neben einem sehr auffälligen Essverhalten (zwanghaftes Streben nach Speisen bei ausbleibendem Sättigungsgefühl) wird sehr oft ein stures Verhalten mit plötzlichen Stimmungsschwankungen und Wutanfällen sowie zwanghaftes Verhalten (Horten von nutzlosen Objekten, das Bestehen auf bestimmten Anordnungen und gleichbleibenden Abläufen, perseverierendes Wiederholen von Fragen) berichtet. Wutanfälle treten oft dann auf, wenn ein Wunsch verweigert oder eine soziale Erwartung nicht erfüllt wird bzw. ein Wechsel gegenüber einer gewohnten Routine gefordert wird.

Zwanghafte Verhaltensweisen treten beim Prader-Willi-Syndrom signifikant häufiger auf als bei Personen mit anderen Formen der intellektuellen Beeinträchtigung. Sie sind schon im frühen Kindesalter zu beobachten, nehmen aber im späteren Jugend- und frühen Erwachsenenalter noch zu. Sie sind nicht assoziiert mit dem Schweregrad der intellektuellen Beeinträchtigung. Diese zwanghaften Verhaltensweisen sind gut vereinbar mit dem Profil der kognitiven Prozesse, das ausgeprägte Schwierigkeiten mit der flexiblen Umstellung der Aufmerksamkeitsrichtung zeigt [30].

## Fazit für die Praxis

- Kinder, Jugendliche und Erwachsene mit intellektueller Beeinträchtigung weisen unterschiedliche kognitive Profile und Auffälligkeiten in den exekutiven Funktionen auf. Sie können – als Verhaltensphänotyp – für bestimmte genetische Syndrome als Ursache der Beeinträchtigung des Verhaltens charakteristisch sein.
- Das Wissen um die Heterogenität bzw. Besonderheiten der kognitiven Profile trägt zu einem besseren Verständnis der Probleme der Personen mit intellektueller Beeinträchtigung bei der Alltagsbewältigung bei. Spezifische Defizite in kognitiven und exekutiven Funktionen erhöhen darüber hinaus im Sinne einer biologischen Disposition die Vulnerabilität für die Ausbildung von Verhaltensauffälligkeiten und psychischen Störungen.
- Für die Praxis bedeutet das, dass die Bestimmung eines globalen Intelligenzquotienten bei der Diagnostik von Kindern, Jugendlichen und Erwachsenen mit intellektueller Beeinträchtigung nicht ausreicht. Vielmehr ist eine differenzierte Beurteilung der kognitiven und exekutiven Funktionen zu empfehlen.
